# HDAC2 Is Involved in the Regulation of BRN3A in Melanocytes and Melanoma

**DOI:** 10.3390/ijms23020849

**Published:** 2022-01-13

**Authors:** Markus V. Heppt, Anja Wessely, Eva Hornig, Claudia Kammerbauer, Saskia A. Graf, Robert Besch, Lars E. French, Alexander Matthies, Silke Kuphal, Melanie Kappelmann-Fenzl, Anja K. Bosserhoff, Carola Berking

**Affiliations:** 1 Department of Dermatology, Universitätsklinikum Erlangen, Friedrich-Alexander University Erlangen-Nürnberg (FAU), 91054 Erlangen, Germany; anja.wessely@uk-erlangen.de (A.W.); carola.berking@uk-erlangen.de (C.B.); 2 Comprehensive Cancer Center Erlangen-European Metropolitan Area of Nuremberg (CCC ER-EMN), 91054 Erlangen, Germany; 3 Department of Dermatology and Allergy, University Hospital, LMU Munich, 80337 Munich, Germany; hornigeva@gmail.com (E.H.); claudia.kammerbauer@med.uni-muenchen.de (C.K.); saskiaannagraf@gmail.com (S.A.G.); Rb@forellen-apo.de (R.B.); lars.french@med.uni-muenchen.de (L.E.F.); 4 Institute of Biochemistry, Friedrich-Alexander University Erlangen-Nürnberg (FAU), 91054 Erlangen, Germany; alexander.matthies@fau.de (A.M.); silke.kuphal@fau.de (S.K.); anja.bosserhoff@fau.de (A.K.B.); 5 Faculty of Computer Science, Deggendorf Institute of Technology, 94469 Deggendorf, Germany; melanie.kappelmann-fenzl@th-deg.de

**Keywords:** melanoma, epigenetic regulation, BRN3A, HDAC, HAT, DNA methylation, ChIP-Seq

## Abstract

The neural crest transcription factor BRN3A is essential for the proliferation and survival of melanoma cells. It is frequently expressed in melanoma but not in normal melanocytes or benign nevi. The mechanisms underlying the aberrant expression of BRN3A are unknown. Here, we investigated the epigenetic regulation of *BRN3A* in melanocytes and melanoma cell lines treated with DNA methyltransferase (DNMT), histone acetyltransferase (HAT), and histone deacetylase (HDAC) inhibitors. DNMT and HAT inhibition did not significantly alter BRN3A expression levels, whereas panHDAC inhibition by trichostatin A led to increased expression. Treatment with the isoform-specific HDAC inhibitor mocetinostat, but not with PCI-34051, also increased BRN3A expression levels, suggesting that class I HDACs HDAC1, HDAC2, and HDAC3, and class IV HDAC11, were involved in the regulation of BRN3A expression. Transient silencing of HDACs 1, 2, 3, and 11 by siRNAs revealed that, specifically, HDAC2 inhibition was able to increase BRN3A expression. ChIP-Seq analysis uncovered that HDAC2 inhibition specifically increased H3K27ac levels at a distal enhancer region of the *BRN3A* gene. Altogether, our data suggest that HDAC2 is a key epigenetic regulator of BRN3A in melanocytes and melanoma cells. These results highlight the importance of epigenetic mechanisms in regulating melanoma oncogenes.

## 1. Introduction

Melanoma ranks among the deadliest types of skin cancer and accounts for the vast majority of deaths from cutaneous neoplasms. It arises from melanocytes, the pigmented cells of the skin. Melanocytes originate from the neural crest, which also gives rise to neurons, glial cells, myocytes, and fibroblasts. During embryonic development, precursor cells located in the neural crest migrate towards the skin where they terminally differentiate into melanocytes [1,2]. In turn, melanoma cells frequently express proteins and reactivate neural crest transcription factors to hijack oncogenic properties such as the ability to migrate and invade into surrounding tissues, resulting in metastatic spread of the disease. Thus, a profound understanding of the neural transcription network may help to develop novel therapeutic strategies in melanoma [3].

BRN3A (encoded by the *POU4F1* gene) is a member of the POU family of transcription factors, which play an important role in the embryonic development of neural crest cells [4]. During embryogenesis, BRN3A is expressed in precursor cells of the peripheral nervous system and early postmitotic neurons of the central nervous system [5], where it acts as an important pro-survival factor of neurons, as demonstrated in knockout mice. BRN3A deficiency leads to the induction of apoptosis in the neurons of the trigeminus and dorsal root ganglions in the late phases of embryonic development, resulting in impaired motility and suckling and subsequent death of the animals soon after birth [6,7]. In adults, BRN3A expression has been found in sensory neurons and parts of the central nervous system as well as in non-neuroectodermal tissues, such as testis, breast, and cervix [8,9,10]. Melanocytes do not express BRN3A; however, it is frequently expressed in melanoma and is essential for the survival of these tumor cells in vitro and in vivo. Its inhibition leads to an arrest in the G1 phase of the cell cycle mediated by p53 and subsequent apoptosis via the intrinsic apoptosis pathway in BRN3A high-expressing melanoma cells but not in melanocytes. This was evident due to the cleavage of caspase 3 and 9 but not of caspase 8. Additionally, antiapoptotic factors such as Bcl-2, Bcl-xL, and Bcl-w levels were reduced, while the proapoptotic Bax and Bak were enhanced after BRN3A depletion in melanoma cells [11]. Ectopic BRN3A expression in melanocytes promotes anchorage-independent growth and reduces oncogene-induced senescence by cooperating with oncogenic BRAF [11]. Besides melanoma, increased BRN3A expression levels have also been described in Ewing sarcoma, neuroepithelioma [9], prostate [12], cervical [13], and ovarian cancer [14], underlining its important role as oncogene.

However, the mechanisms underlying the aberrant expression of BRN3A in melanoma are widely unknown. Gene expression is normally tightly controlled and modulated by different DNA modifications and chromatin remodeling mechanisms. Gene silencing is mostly mediated by cytosine hypermethylation of CpG islands in the promoter regions through DNA methyltransferases (DNMTs), resulting in strong downregulation of gene expression [15,16,17]. Besides DNA methylation, a more dynamic method of gene regulation is mediated by histone modifications involving the acetylation, methylation, and phosphorylation at different sites of the histone tails [18]. Acetylation of lysine residues at N-terminal histone tails by histone acetyltransferases (HATs) decreases the binding affinity of histones and DNA and leads to chromatin decondensation. Thereby, the DNA globally becomes more accessible for transcription factors, resulting in increased gene transcription. For the downregulation of genes, histone deacetylases (HDACs) remove the acetyl residues to restore the tight interaction between DNA and histones [18,19]. It is estimated that HDACs control the expression of approximately 2 to 10% of all genes [20]. Today, 18 members and 4 classes of HDACs have been discovered so far. HDAC1, HDAC2, HDAC3, and HDAC8 belong to the best-studied class I HDACs and are involved in the modulation of gene expression, cell cycle progression, and DNA damage control [21]. Yet, their involvement in the regulation of melanoma oncogenes is little established.

In this study, we investigated epigenetic mechanisms that are involved in the regulation of the *BRN3A* expression in melanocytes and melanoma cells. Using a pharmacologic and gene-specific approach, we have identified class I histone deacetylase 2 (HDAC2) as a key epigenetic regulator of *BRN3A* in melanocytes. Furthermore, we propose an annotated enhancer region of *BRN3A* that was specifically hyperacetylated after inhibition of HDAC2.

## 2. Results

### 2.1. BRN3A Expression in Melanocytes Is Not Silenced by DNA Hypermethylation

**Figure 1:**
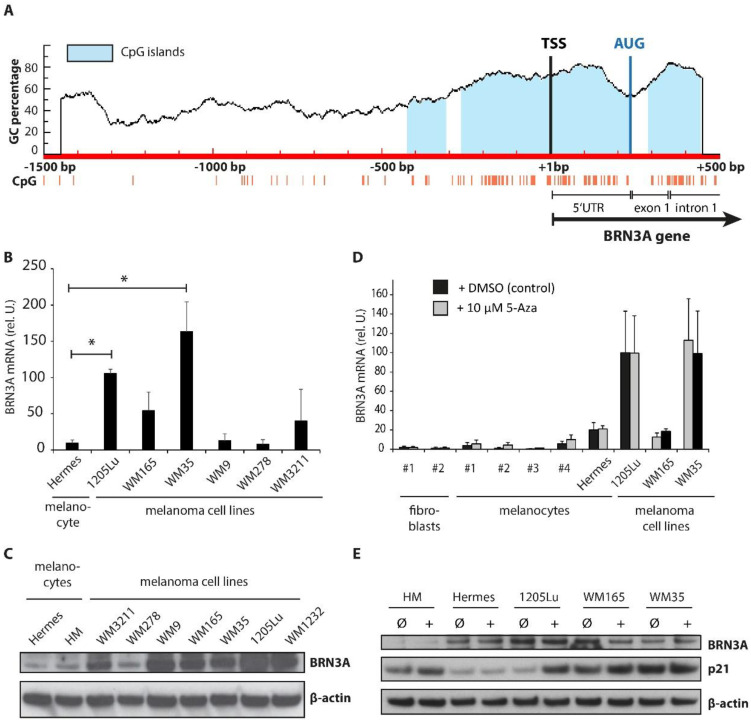
BRN3A expression in melanocytes is not controlled by DNA methylation. (**A**) In silico analysis of the *BRN3A* promoter region from −1500 bp upstream and +500 bp downstream of the transcription start site (TSS, position: +1 bp). CpG dinucleotides are depicted as orange lines, and predicted CpG islands are shown in light blue. Methylation status of CpGs located within the region from position −229 bp to −11 bp upstream of the *BRN3A* TSS was also analyzed by bisulfite sequencing (see also Supplementary Figure S1A). AUG: start codon of *BRN3A* (position: +235 bp downstream of the *BRN3A* TSS). (**B**) Basal gene and (**C**) protein expression levels of BRN3A in immortalized human melanocytes (Hermes) and a panel of melanoma cell lines (1205Lu, WM165, WM35, WM9, WM278, WM3211, and WM1232), n = 3, *: *p* < 0.05 vs. Hermes. (**D**) Relative *BRN3A* gene expression in human fibroblasts (HF, 2 donors), melanocytes (HM, 4 donors), Hermes cells, and a panel of melanoma cell lines (1205Lu, WM165, and WM35) after treatment with 10 µM 5-Aza-2′deoxycytidine (5-Aza) or DMSO (control) for 48 h, n = 3. (**E**) Protein expression of BRN3A and the cell cycle regulating protein p21^Cip1/Waf1^ (p21) after treatment with 10 µM 5-Aza (+) or DMSO (control, Ø) for 48 h. One representative immunoblot is shown.

DNA hypermethylation of CpG islands is a common mechanism to effectively silence gene expression [15]. To identify potential CpG islands in the promoter region of the *BRN3A* gene, an in silico analysis of this region was conducted. The *BRN3A* promoter region between 1500 bp upstream and 500 bp downstream of the transcription start site (TSS), including the 5’ untranslated region (5′UTR), exon 1, and parts of the first intron, was screened for CpG islands using the *MethPrimer* software [22,23]. Interestingly, CpG islands were abundant within these regions. Three CpG islands were identified: island 1 reaching from −424 bp to −308 bp upstream of the *BRN3A* TSS (spanning 117 bp), island 2 from −264 bp to +235 bp (499 bp), and island 3 from +293 bp to +444 bp (152 bp), covering parts of exon 1 and intron 1 (Figure 1A). Thus, these CpG islands were potential target sites for DNA hypermethylation, which may lead to permanent silencing of *BRN3A* in melanocytes. We used a pharmacological approach to investigate if DNA demethylation leads to upregulation of BRN3A in melanocytes. Therefore, human melanocytes (HM, up to four different donors), immortalized human melanocytes (Hermes), and cutaneous melanoma cell lines, with low, moderate, and high basal BRN3A expression levels representing distinct growth stages, as well as human fibroblasts (HF, two donors, without basal BRN3A expression) (Figure 1B,C), were treated with the DNMT inhibitor 5-Aza-2′deoxycytidine (5-Aza, decitabine) for 48 h. 5-Aza is a cytidine analog that is incorporated into the DNA during cell replication. DNMTs methylate the newly synthesized DNA according to the methylation pattern of the paternal DNA strand to maintain the DNA methylation status. During this methylation step, incorporated 5-Aza irreversibly forms covalent bonds with the active sites of DNMTs, resulting in a passive DNA demethylation [24]. Thus, longer incubation times and proliferating cells are required to achieve sufficient DNA demethylation by pharmacologic DNMT inhibition. Gene and protein expression analysis revealed no induction of BRN3A by 5-Aza in HM, HF, or any tested melanoma cell line, suggesting that DNA hypermethylation did not play a major role in the regulation of BRN3A expression (Figure 1D,E). We also analyzed the gene and protein expression levels of the cell cycle regulating protein p21^Cip1/Waf1^ (p21) as well as 14-3-3 σ (stratifin, SFN), to evaluate the effects of the DNMT inhibition. The promoter of the *SFN* gene encoding the 14-3-3 σ protein is hypermethylated in melanocytes and melanoma cells, and p21 expression can be upregulated by 5-Aza treatment in melanoma cells, as shown previously [25,26,27]. We observed a strong induction of *SFN* gene expression and a moderate induction of the *CDKN1A* gene encoding p21 upon 5-Aza treatment in almost all tested cells (Supplementary Figure S1B,C). Protein expression levels of p21 were increased markedly in 1205Lu (Figure 1E), and to a lesser extent in melanocytes, Hermes, WM165, and WM35 (Supplementary Figure S1D,E).

To confirm the results of the DNMT inhibition experiments, we also analyzed the basal methylation status of the proximal *BRN3A* promoter region in HM; Hermes; and the melanoma cell lines 1205Lu, WM165, and WM35. Genomic DNA was isolated, and unmethylated cytosines were converted to uracil by bisulfite treatment. The genomic region from position −229 bp to −11 bp upstream of the *BRN3A* TSS (Figure 1A), which contains a putative CpG island as predicted by the *MethPrimer* software [22,23], was chosen for PCR amplification and subsequent Sanger sequencing for methylation status analysis. Interestingly, all cytosines in this genomic region were converted to uracil by bisulfite treatment, indicating that none of the CpG dinucleotides were initially methylated and thereby protected from bisulfite conversion (Supplementary Figure S1A). Altogether, these data strongly suggest that the expression of *BRN3A* is not silenced by DNA hypermethylation.

### 2.2. Influence of HAT Inhibition on BRN3A Expression Levels

In addition to DNA modifications, several forms of histone modifications play an important role in the dynamic regulation of gene expression. Histone acetylation contributes significantly to the chromatin condensation status and is controlled by histone acetyltransferases (HATs) and histone deacetylases (HDACs) as their counterparts [18,19].

**Figure 2:**
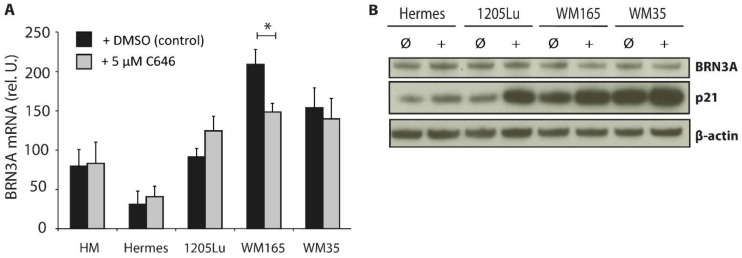
BRNA expression levels after HAT inhibition. (**A**) Relative *BRN3A* gene expression in HM, Hermes, and melanoma cell lines (1205Lu, WM165, and WM35). Cells were incubated with 5 µM C646 or DMSO (control) for 6 h, n = 3; *: *p* < 0.05 vs. corresponding DMSO control. (**B**) Protein expression of BRN3A and p21 after treatment with 5 µM C646 (+) or DMSO (control, Ø) for 24 h; one representative immunoblot is shown.

To investigate the role of histone acetylation in *BRN3A* expression regulation, HM, and Hermes, a panel of melanoma cells were treated with the p300 histone acetyltransferase inhibitor C646 [28]. After HAT inhibition, the BRN3A expression levels decreased slightly in one tested melanoma cell line (WM165) (Figure 2A,B). C646 treatment increased the p21 expression levels as described previously by [29] in all investigated cell lines, indicating that HAT inhibition was successful (Supplementary Figure S1E).

### 2.3. Trichostatin A Induces BRN3A Expression in Melanocytes and Melanoma Cell Lines

**Figure 3:**
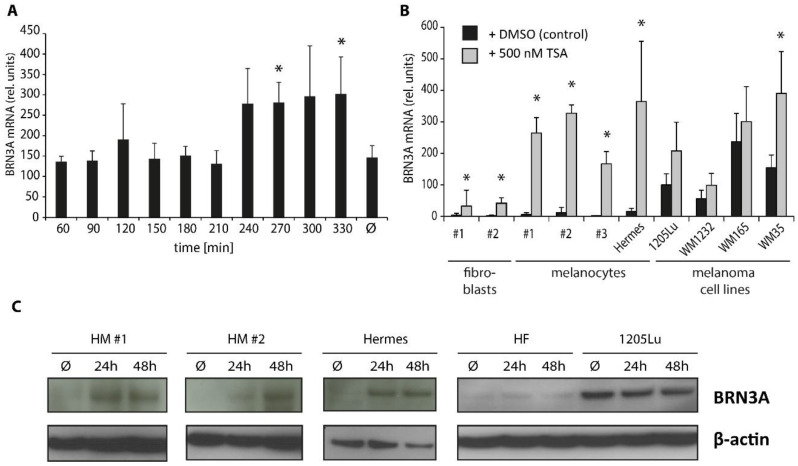
TSA induces BRN3A expression in melanocytes and melanoma cells. (**A**) Timeline analysis of *BRN3A* gene expression by qRT-PCR in human melanocytes (HM). HM were incubated with 500 nM TSA for up to 6 h. 0 min: before treatment, Ø: DMSO (control) after 6 h incubation. *: *p* < 0.05 vs. DMSO control, n = 3. (**B**) Relative *BRN3A* gene expression after panHDACi by TSA. Human fibroblasts (2 donors), melanocytes (3 donors and Hermes), and melanoma cell lines (1205Lu, WM1232, WM165, and WM35) were incubated with 500 nM TSA or DMSO (control) for 6 h. *: *p* < 0.05 vs. DMSO control, n = 3. (**C**) Protein expression of BRN3A in melanocytes (2 donors and Hermes), human fibroblasts (HF), and one melanoma cell line (1205Lu) after treatment with 500 nM TSA or DMSO (Ø, control) for 24 h and 48 h, respectively. Representative immunoblots are shown.

Considering the slight decrease of BRN3A after HAT, we next focused on HDACs as possible epigenetic regulators and tested the impact of the highly potent broad-spectrum HDAC inhibitor (panHDACi) trichostatin A (TSA) [30] on *BRN3A* expression levels. In a timeline analysis conducted in HM, TSA treatment increased *BRN3A* gene expression significantly in a time-dependent manner. The highest levels were observed after 4.5 h and 6 h of TSA treatment (Figure 3A), suggesting that TSA directly influenced the regulation of the *BRN3A* gene. Gene expression analysis in melanocytes and melanoma cell lines after 6 h revealed that TSA strongly induced the expression levels in HM, Hermes, and one tested melanoma cell line (WM35) (Figure 3B). After 24 h and 48 h, we detected elevated BRN3A protein expression levels in HM and Hermes but not in melanoma cells, probably due to the already high basal BRN3A expression, which could not be further enhanced by TSA (Figure 3C). In human skin fibroblasts (HF), a slight increase of *BRN3A* gene expression but not of BRN3A protein levels was observed upon HDAC inhibition (Figure 3B,C).

TSA treatment for more than 24 h led to extended cell death in both Hermes and melanoma cells, as reported previously [31] (Supplementary Figure S2A,B). Therefore, further functional assays that require longer incubation times were not possible. In summary, panHDAC inhibition by TSA increased the expression of *BRN3A* in low-expressing normal and immortalized melanocytes, indicating that HDACs play a major role in the epigenetic regulation of *BRN3A*.

### 2.4. Class I HDAC Inhibition Increases BRN3A Expression in Melanocytes

**Figure 4:**
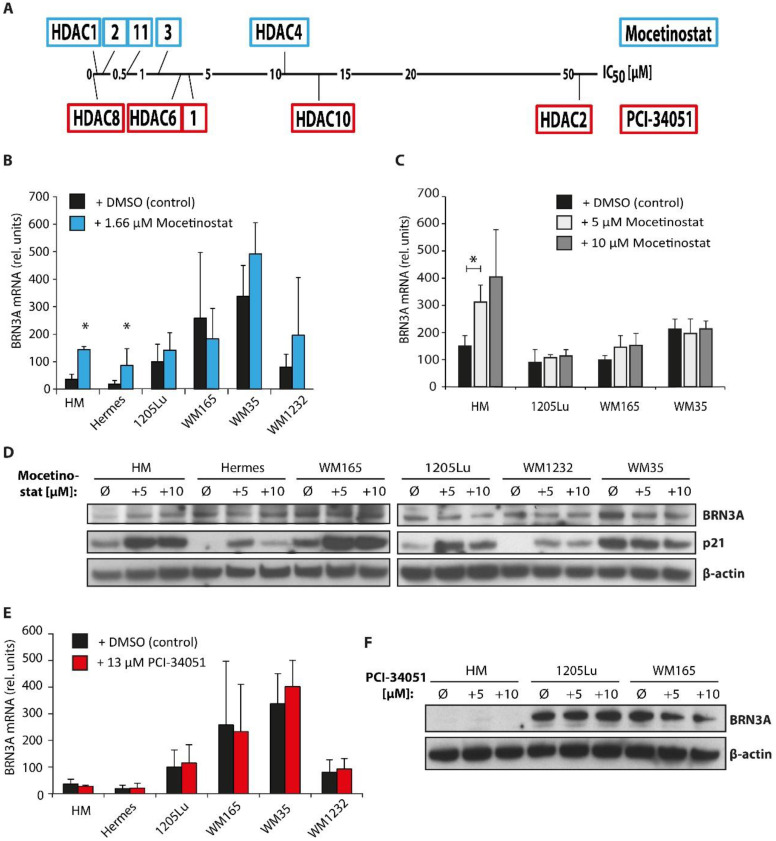
Inhibition of class I HDACs induced BRN3A expression in melanocytes. (**A**) Schematic overview of IC50 values of selective HDAC inhibitors mocetinostat (blue box) and PCI-34051 (red box) for different HDACs. In concentrations below 20 µM, mocetinostat inhibits HDAC1, 2, 3, and 11, whereas PCI-34051 inhibits HDAC1, 6, 8, and 10 [32,33]. (**B**) Relative *BRN3A* gene expression levels in melanocytes (HM, Hermes) and melanoma cells after treatment with 1.66 µM mocetinostat or DMSO (control) for 6 h, n = 3. *: *p* < 0.05 vs. corresponding DMSO control. (**C**) Relative *BRN3A* gene expression levels in melanocytes and melanoma cells after treatment with 5 µM or 10 µM mocetinostat or DMSO (control) for 6 h, n = 3. *: *p* < 0.05 vs. corresponding DMSO control. (**D**) BRN3A protein expression in melanocytes (HM, Hermes) and melanoma cells after treatment with 5 µM (+ 5) and 10 µM (+ 10) mocetinostat for 24 h. Ø: DMSO (control), representative immunoblot. (**E**) Relative *BRN3A* gene expression levels in melanocytes (HM, Hermes) and melanoma cells after treatment with 13 µM PCI-34051 or DMSO (control) for 6 h, n = 3. (**F**) BRN3A protein expression in HM and melanoma cells after treatment with 5 µM (+ 5) and 10 µM (+ 10) PCI-34051 for 24 h. Ø: DMSO (control); one representative immunoblot is shown.

Next, we used more selective pharmacological HDAC inhibitors to narrow down the candidates that were potentially involved in the regulation of BRN3A. HM, Hermes, and several melanoma cell lines were treated with the HDAC inhibitors mocetinostat or PCI-34051 (for IC_50_ values see Figure 4A), and BRN3A expression was determined. Mocetinostat predominantly inhibits class I HDACs HDAC1, HDAC2, HDAC3, and HDAC11, the only class IV member [32]. PCI-34051 inhibits the class I HDACs, HDAC1, HDAC8, and HDAC6, and at higher concentrations also HDAC10 (both class IIb HDACs) [33]. Low concentrations of mocetinostat (c = 1.66 µM) significantly increased *BRN3A* gene expression in HM and Hermes (Figure 4B). Higher concentrations of mocetinostat (5 µM and 10 µM) further increased *BRN3A* gene expression levels in melanocytes in a dose-dependent manner (Figure 4C). However, PCI-34051 (c = 13 µM) did not alter *BRN3A* gene expression levels in any investigated cell line (Figure 4E). Similar to panHDACi by TSA, upregulation of *BRN3A* by mocetinostat was already detectable after 6 h. Immunoblot analysis confirmed increased BRN3A expression in HM by mocetinostat but not by PCI-34051 in a dose-dependent manner (Figure 4D,F), indicating that class I HDACs and, especially, HDAC2, HDAC3, and HDAC11, were crucially involved in the epigenetic *BRN3A* expression regulation in melanocytes. In melanoma cells, no alterations of the *BRN3A* expression levels were observed.

### 2.5. HDAC2 Is a Key Epigenetic Regulator of BRN3A in Melanocytes

**Figure 5:**
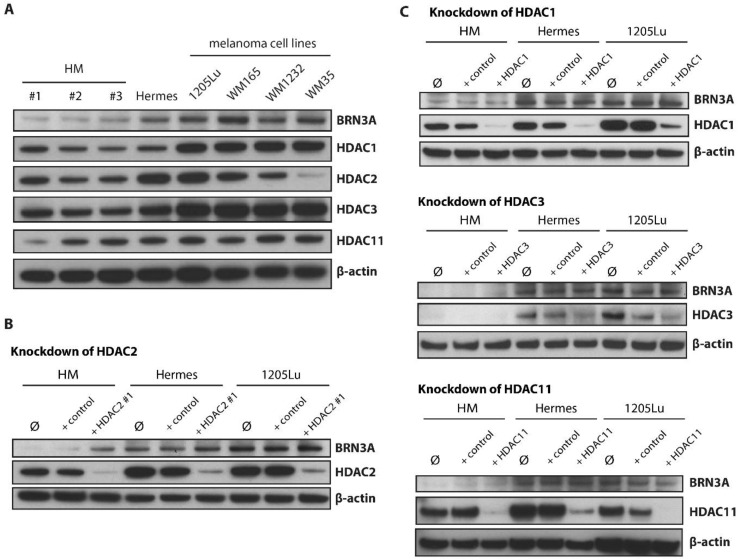
HDAC2 inhibition induced BRN3A expression in melanocytes and melanoma cells. (**A**) Basal protein expression of HDAC1, HDAC2, HDAC3, and HDAC11, in HM (3 donors), Hermes, and melanoma cell lines (1205Lu, WM165, WM1232, and WM35). (**B**) Protein expression of BRN3A and HDAC2 48 h after siRNA-mediated transient knockdown of HDAC2 in melanocytes (HM, Hermes) and melanoma cells (1205Lu). HDAC2 #1: HDAC2 siRNA. BRN3A induction upon HDAC2 inhibition was also confirmed by a second siRNA (HDAC2 #2, see Supplementary Figure S3A); control: non-targeting control siRNA; Ø: transfection reagent only (HM, Hermes: JetPrime; 1205Lu: Lipofectamine RNAiMAX). (**C**) Protein expression of BRN3A as well as HDAC1, HDAC3, and HDAC11 48 h after knockdown of HDAC1, 3, and 11, respectively, in melanocytes (HM, Hermes) and melanoma cells (1205Lu). HDAC1, HDAC3, and HDAC11: specific siRNAs targeting HDAC1, 3, and 11, respectively; control: non-targeting control siRNA; Ø: transfection reagent only (HM, Hermes: JetPrime; 1205Lu: Lipofectamine RNAiMAX). Representative immunoblots are shown.

Basal protein expression levels of BRN3A, as well as HDAC1, HDAC2, HDAC3, and HDAC11, which were inhibited by mocetinostat were determined (Figure 5A). To identify specific HDACs members, which may act as critical regulators of *BRN3A* expression, we used siRNAs to inhibit HDAC1, HDAC2, HDAC3, and HDAC11 selectively in a gene-specific approach. Surprisingly, only HDAC2 inhibition with two different siRNAs was able to increase BRN3A protein expression in HM and, to a lesser extent, in Hermes cells (Figure 5B and Supplementary Figure S3A,B). HDAC1 inhibition did not affect BRN3A expression levels as might have been expected due to its homology to HDAC2 [34]. Neither HDAC3 nor HDAC11 inhibition induced the expression of BRN3A in any investigated cell line (Figure 5C). Ectopic overexpression of FLAG^®^-tagged HDAC2 in BRN3A high-expressing 1205Lu and WM165 cells did not result in a downregulation of BRN3A, suggesting that other factors are involved (Supplementary Figure S3C).

To investigate if HDAC2 inhibition alters the acetylation of its target histones at DNA regulatory elements that specifically regulate *BRN3A*, we mapped the binding regions of acetylated lysine 27 residues at histone H3 (H3K27ac) using chromatin immunoprecipitation sequencing (ChIP-Seq). ChIP-Seq experiments after HDAC2 inhibition were performed using the melanoma cell line WM9 with low basal *BRN3A* gene expression levels instead of melanocytes, due to technical issues (sufficient cell number, high grade of pigmentation). The low mRNA levels are in contrast to high endogenous protein expression of BRN3A in this line (Figure 1C), which is consistent with previous studies and might be due to a negative feedback loop or posttranslational modifications of BRN3A protein [11]. This cell line showed significantly increased *BRN3A* mRNA and protein expression levels as soon as 24 h after siRNA-mediated HDAC2 inhibition and mocetinostat treatment (Supplementary Figure S3D–F).

**Figure 6:**
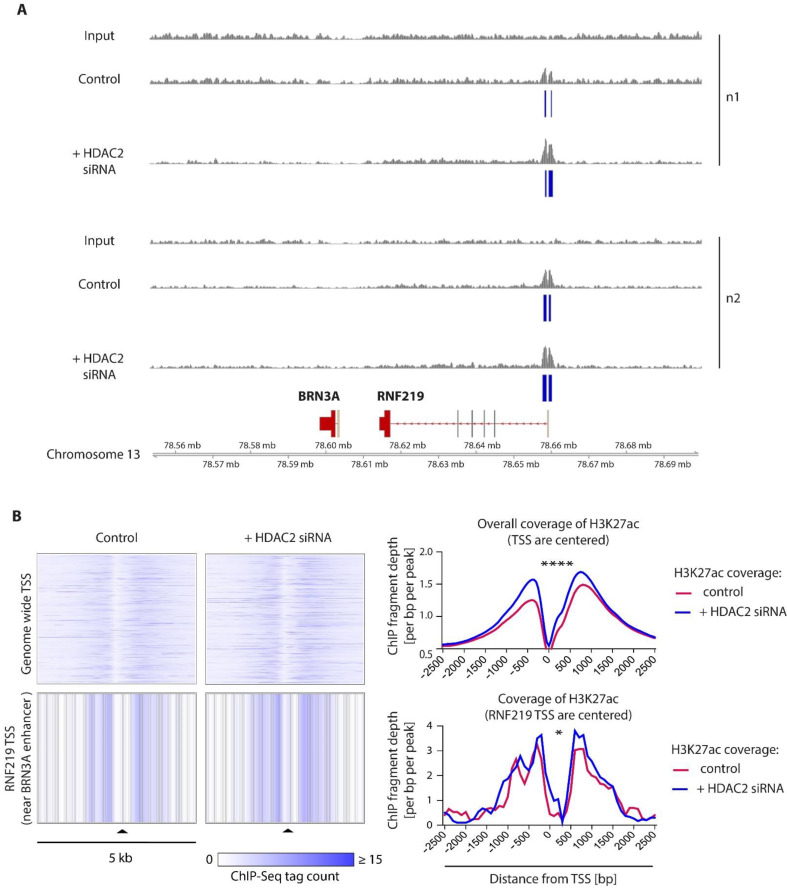
HDAC2 inhibition increases H3K27 acetylation (H3K27ac) status in a distal enhancer element of *BRN3A*. (**A**) H3K27 acetylation status of the genetic region chr13:78,550,000–78,700,000 (hg38) in WM9 melanoma cells transfected with HDAC2-specific siRNA or control siRNA (control) compared to input. Peaks illustrate the normalized read counts. Blue bars highlight identified peaks with a false discovery rate of 1 × 10^−6^ compared to input in the genomic region chr13:78,656,679–78,661,679, which is located near to the TSS of the *RNF219* gene and annotated as a promoter/enhancer element of *BRN3A* in the GeneHancer database (GeneHancer Identifier GH13J078657, https://www.genecards.org/cgi-bin/carddisp.pl?gene=POU4F1, accessed on 18 September 2019 [37]). (**B**) Heat maps and histograms of ChIP-Seq tag counts of the H3K27ac status in a 5 kb wide range around overall TSS and the *RNF219* TSS. Profiles show a significant increase of H3K27ac rate around global (****: *p* = 0.0001; Wilcoxon test, paired, two-sided) and the *RNF219* TSS (*: *p* = 0.0156; Wilcoxon test, paired, two-sided), representing the *BRN3A* enhancer element GH13J078657.

HDAC2 was silenced by transfection with specific siRNAs in WM9 cells, then the histones were cross-linked and incubated with antibodies targeting H3K27ac, a target of HDAC2 and epigenetic modification that is associated with active enhancers [35]. Strikingly, ChIP-Seq analysis using the melanoma cell line WM 9 revealed that HDAC2 inhibition leads to an enrichment of H3K27ac at one specific promoter/enhancer element that is located 55.4 kb upstream of the *BRN3A* TSS within the proximal promoter of the *RNF219* gene. This enhancer has been annotated as GH13J078657 in the GeneHancer database as a specific enhancer of *BRN3A* (accessible via GeneCards, https://www.genecards.org/cgi-bin/carddisp.pl?gene=POU4F1, accessed on 18 September 2019 [36]) (Figure 6A,B).

In summary, our results show that pharmacological inhibition of class I HDACs by TSA and mocetinostat is sufficient to induce *BRN3A* gene expression in melanocytes. In particular, we narrowed down the potential HDAC candidates by targeting single HDACs with siRNAs and identified HDAC2 as a crucial regulator of *BRN3A* gene expression. Furthermore, the ChIP-Seq results suggest that HDAC2 influences *BRN3A* gene expression levels via a distal enhancer element annotated as GH13J078657 in the GeneHancer database.

## 3. Discussion

The neural crest transcription factor BRN3A plays a pivotal role in the survival of melanoma cells [11]. However, the reasons for its aberrant expression in melanoma but not in melanocytes or benign melanocytic nevi have not been elucidated. In this study, we investigated several epigenetic regulatory mechanisms and their contribution to BRN3A expression in melanocytes and a panel of melanoma cell lines. The selection of the melanoma cell lines was primarily guided by (i) the expression levels of BRN3A determined by a previous study and (ii) to reflect the distinct growth phases of the classic disease sequence model of melanoma [11,38]. The latter model defines several stages such as radial growth phase (RGP), vertical growth phase (VGP), and metastatic lines. The core experiments in this study were performed in the three cell lines WM165 (moderate BRN3A, metastatic), WM35 (moderate to high BRN3A, RGP), and 1205Lu (moderate to high BRN3A, metastatic). The cell lines WM9 (metastatic) and WM1232 (RGP) both showed low levels of mRNA but moderate levels of BRN3A protein. As we supposed that epigenetic mechanisms would affect gene expression, we used those lines as representatives for low BRN3A expression. HDAC2 was identified as a key epigenetic regulator of *BRN3A* gene expression in melanocytes, and HDAC2 inhibition led to an increase in H3K27 acetylation in a distal enhancer element of *BRN3A*. Thus, our results highlight the importance of epigenetic regulatory mechanisms in the malignant progression from melanocytes to melanoma.

HDAC2 is a member of the class I HDAC family. These enzymes are ubiquitously expressed and predominantly located in the nucleus of the cell, where they contribute to chromatin condensation and downregulation of gene expression by removing acetyl groups from lysine residues at the N-terminal histone tails [18,39]. HDAC2 and the closely related HDAC1 share 85% identity in protein sequence [34]. As a consequence, HDAC2 and HDAC1 are often regarded as functionally redundant, and HDAC2 knockdown can be frequently compensated for by HDAC1 functional activity and vice versa [40]. Therefore, it would not have been surprising if HDAC1 knockdown led to similar results to HDAC2 knockdown in our experiments. However, only HDAC2 but not HDAC1 was involved in the regulation of BRN3A as HDAC1 inhibition did not alter BRN3A expression levels. Thus, HDAC2 but not HDAC1 seems to be the critical HDAC that influences the BRN3A expression in melanocytes and melanoma cells with low endogenous mRNA expression of *BRN3A*.

Over the last few years, aberrant epigenetic modifications and their contribution to tumor formation, progression, and prognosis have been studied extensively [41]. Deregulated HDAC expression has been reported in a variety of human cancers, and several HDAC inhibitors are currently under investigation in clinical trials. In particular, HDAC2 overexpression has been described in ovarian cancer [42] and has been associated with aggressive cutaneous T cell lymphoma [43], advanced-stage gastric [44], colorectal cancer [45], and shorter disease-free survival of prostate cancer patients [46]. In preclinical studies, HDAC inhibitors such as vorinostat, entinostat, and panobinostat have shown promising anti-cancer effects in several cancer entities, including melanoma [20,21]. In this entity, HDAC inhibition affects the cell cycle, survival, proliferation, immunogenicity, and resistance to targeted therapies in vitro and in vivo. These studies suggest that HDACs can contribute significantly to the malignancy of melanoma, although the role of single HDAC proteins is poorly understood [21]. Class I HDAC inhibition increases the expression of PD-L1 and PD-L2 in melanoma cells, providing the rationale for combining HDAC inhibitors and immune checkpoint blocking antibodies [47]. Additionally, the expression of tumor-associated antigens such as MHC class I and class II molecules, as well as costimulatory CD80 and CD40, is also augmented by HDAC inhibition, so these tumors get more susceptible for immunotherapy [48]. Similarly, HDAC inhibition leads to increased expression of HLA class I molecules as well as MHC class I chain-related protein (MIC) A and B in Merkel cell carcinoma, another highly aggressive skin cancer [49,50], highlighting the important role of epigenetic regulators for the immune therapy of skin cancer.

Increased expression of class I HDACs has been reported in cell lines derived from patients with BRAF inhibitor-resistant melanoma [51], indicating that HDACs are possibly involved in the development of acquired resistance to BRAF inhibitors. Despite the promising results in preclinical studies, the first clinical trials exploring HDAC inhibitors as monotherapy were quite disappointing, with only modest anti-tumor activity in most investigated malignancies [52]. However, therapies combining HDAC inhibitors with additional anti-cancer drugs as chemotherapeutics or targeted therapies seem to be more attractive, as synergistic or additive anti-tumor effects have been reported for several cancer entities in preclinical studies as well as in phase I clinical trials [20,53,54]. These observations demonstrate that HDAC regulation in cancer and its contribution to therapy resistance and tumor progression might be more complex than it seems at first sight. HDACs exert pleiotropic effects, which may limit their therapeutic potential. Here, understanding specific regulatory pathways of established oncogenes can help to develop a more targeted approach for epigenetic treatments.

Expression and correlation with disease stage or prognosis of HDAC proteins in melanoma have not been studied to date. We detected moderate to high global HDAC2 expression levels in a panel of melanoma cells and no expression or only relatively low levels in melanocytes. In almost all investigated melanoma cell lines, high basal HDAC2 expression seemed to be correlated with lower basal BRN3A expression and *vice versa*. However, the effects on BRN3A expression through pharmacologically and siRNA-mediated HDAC inhibition did not strictly correlate with the overall HDAC2 protein expression levels. HDAC2 inhibition in cells with high basal expression levels did not result in a strong induction of the BRN3A expression. It is conceivable that the HDAC2 overall protein expression levels do not necessarily correlate with the HDAC2 activity at one specific locus of the genome (e.g., *BRN3A* promoter or enhancer region), and further investigations are needed to address this question more precisely. In this context, it is notable that HDAC inhibition only led to an increase in BRN3A expression levels in cells with a relatively low basal *BRN3A* mRNA expression, such as WM9. We hypothesize that *BRN3A* expression levels cannot be further enhanced by HDAC inhibition in melanoma cell lines with high intrinsic *BRN3A* levels, such as WM165 or 1205Lu. Our findings suggest that HDAC2 is not the sole regulator of BRN3A and that additional specific interaction partners or transcriptional regulators are likely to be involved in the recruitment of HDAC2 to the *BRN3A* gene locus and its aberrant expression in melanoma. This idea is also supported by the fact the BRN3A levels did not decrease after ectopic expression of HDAC2.

ChIP-Seq analysis for H3K27ac, an epigenetic modification that is associated with active DNA regulatory elements [35], revealed that HDAC2 inhibition did not increase H3K27ac in the genomic region immediately around the *BRN3A* TSS but in a region located 55.4 kb upstream. This region, named GH13J078657 in the GeneHancer database, is annotated as a distal enhancer of *BRN3A* [36,37]. The GeneHancer database contains unbiased data of more than 280,000 DNA regulatory elements as promoters and enhancers mined from the databases ENCODE, Ensembl, the FANTOM project, and the VISTA Enhancer Browser and provides valuable information on enhancer–gene associations [37]. In this database, the element GH13J078657 is classified as an “elite enhancer”, indicating a strong potential relationship between *BRN3A* and GH13J078657 [37]. Interestingly, HDAC2 is listed among the factors that have potential binding sites in this genomic region. Together with a fast induction of *BRN3A* gene expression as observed in the experiments with the pharmacological HDACi TSA and mocetinostat, data from the ChIP-Seq analysis suggested that HDAC2 modulates *BRN3A* expression directly through this enhancer element. However, other transcription factors and transcriptional regulators may also bind to GH13J078657 and contribute to *BRN3A* expression regulation; GeneHancer reports potential binding sites of 164 other transcription factors and regulators in this genomic region [36]. The involvement of other factors might also explain why ectopic HDAC2 expression did not result in a decrease of BRN3A expression in our experiments. Additionally, the so far only poorly described proximal promoter region might also play a role in the regulation of *BRN3A* but is left unaltered by histone acetylation. Therefore, future studies are needed to dissect the complex mechanisms by which BRN3A expression is upregulated in melanoma. Furthermore, it is interesting to notice that GH13J078657 shares its genomic location with the proximal promoter of Ring Finger Protein 219 (*RNF219*, also described as OBI1), which is involved in the regulation of DNA replication [55]. RNF219 acts as a ubiquitin ligase of ORC3/ORC5 proteins that are part of the ORC multi-protein complex, which recognizes origins of replication during the S-phase of the cell cycle. Recent data from Coulombe et al. suggest that RNF219 is essentially required for replication origin selection during DNA replication [55]. Apart from that, little is known about its biological function, and its role in cancer formation and progression has not been elucidated yet.

Besides histone acetylation, we also focused on other epigenetic modifications that may influence gene expression. We used a pharmacologic approach to evaluate the contribution of DNA methylation to *BRN3A* gene silencing in melanocytes and other cell types. Recently, promoter hypomethylation was identified in melanoma cells as the epigenetic cause for the overexpression of Deleted in Split hand/Split foot 1 (DSS1), a tumor-promoting gene that is involved in DNA double-strand repair [56]. Similar to this finding, increased BRN3A levels in melanoma could also be attributed to DNA hypomethylation of its promoter. The in silico analysis showed three predicted CpG islands within the *BRN3A* promoter region that could be putative target sites for DNA methylation. However, DNMT inhibition by 5-Aza did not alter the BRN3A expression levels in any tested cell types. This was also the case for fibroblasts that do not express BRN3A. Additionally, bisulfite conversion and subsequent sequencing of the putative CpG island located upstream of the TSS revealed that none of the cytosines in this genomic region were methylated. Based on these findings, the low BRN3A expression levels in melanocytes are unlikely to be caused by DNA hypermethylation and subsequent gene silencing.

Interestingly, we observed a strong induction of BRN3A after HDAC2 inhibition in normal human melanocytes. This finding might become relevant for therapeutic HDAC inhibition, regarding potential melanocyte-specific adverse effects of these drugs, as BRN3A has been shown to promote melanocytic transformation and tumorigenesis [11]. Most of the HDAC inhibitors that are currently under investigation in clinical trials or have not been approved yet, such as panobinostat (approved for the therapy of multiple myeloma in 2015 by the EMA [57]), inhibit a broad variety of HDACs including HDAC2. Therefore, it would be interesting to investigate if malignant transformation of melanocytes occurs more often in patients receiving HDAC inhibitors. This would also be an interesting question to address in in vitro studies. However, for sufficient BRN3A induction, HDAC inhibitors are required at relatively high concentrations; they also lead to cell death after prolonged incubation, as described previously [31]. Thus, HDAC inhibition might lead to increased BRN3A protein levels that may promote survival on the one hand, but on the other hand, strong toxic effects counteract the high expression of survival-promoting BRN3A and lead to extended cell death.

## 4. Materials and Methods

### 4.1. Cell Culture

Human melanoma cell lines were kindly provided by Meenhard Herlyn (Wistar Institute, Philadelphia, PA, USA) and cultivated in TU2% medium containing 80% MCDB-153 (Sigma-Aldrich, Taufkirchen, Germany), 20% Leibovitz’s L-15 (Gibco by Life Technologies, Carlsbad, CA, USA), 2% fetal bovine serum (Biochrom, Berlin, Deutschland), 5 µg/mL bovine insulin, and 1.68 mM CaCl_2_ (both Sigma-Aldrich, Taufkirchen, Germany) at 37 °C with 5% CO_2_. Primary human melanocytes, immortalized human melanocytes (Hermes), and human fibroblasts were isolated from human foreskins or purchased by Lifeline Cell Technology (Walkersville, MD, USA) and cultured as described previously [11,58,59]. Melanoma cell lines were authenticated by STR profiling (Cell Line Authentication Service of Eurofins Genomics, Ebersberg, Germany), and all cells were regularly tested for mycoplasma contamination with the Venor^TM^ GeM Mycoplasma Detection Kit (Minerva Biolabs, Berlin, Germany). For experiments, 85,000 (melanoma cells) or 100,000 cells (HM, Hermes, HF) per well were seeded in six-well plates one day prior to transfection with siRNAs, expression plasmids, or incubation with inhibitors.

### 4.2. Pharmacologic DNMT, HAT, and HDAC Inhibition

For pharmacologic inhibition experiments, the DNMT-inhibitor 5-Aza-2’-deoxycytidine (decitabine, from Sigma-Aldrich, Taufkirchen, Germany), HAT inhibitor C646 (Selleckchem, Munich, Germany), and the HDAC inhibitors trichostatin A (Sigma-Aldrich Taufkirchen, Germany), mocetinostat and PCI-34051 (both Selleckchem, Munich, Germany) were purchased as ready-to-use solutions (TSA), or stock solutions were prepared by dissolving the powder in DMSO (Sigma-Aldrich, Taufkirchen, Germany) according to the manufacturers’ instructions. DMSO (Sigma-Aldrich, Taufkirchen, Germany) was also used for negative controls. Working solutions of inhibitors in cell-specific medium were freshly prepared immediately prior to use. The following concentrations were used: 5-Aza: 10 µM; C646: 5 µM; TSA: 500 nM; mocetinostat: 1.66 µM, 5 µM, and 10 µM; and PCI-34051: 13 µM, 5 µM, and 10 µM. For gene expression analysis, RNA was isolated after 6 h (C646 and all HDACi) or 48 h (5-Aza) incubation. For protein expression analysis, cells were harvested after 24 h (C646, all HDACi) or 48 h (TSA, 5-Aza). As 5-Aza is highly unstable in aqueous solutions, medium containing 5-Aza was changed in all DNMT inhibition experiments after 24 h.

### 4.3. RNA Extraction and Quantitative Real-Time PCR (qRT-PCR)

Total RNA was extracted with the RNeasy Mini Kit (Qiagen, Hilden, Germany) according to the manufacturer’s instructions. In experiments investigating *SFN* gene expression coding for 14-3-3 σ, a DNase digestion step (Qiagen Hilden, Germany) was included during RNA isolation in order to minimize DNA contaminations as no intron-spanning primers could be designed for this gene. One µg RNA was reversely transcribed with Expand Reverse Transcriptase (Roche, Penzberg, Germany) and poly(dT) oligonucleotide primers (Eurofins Genomics, Ebersberg, Germany). Quantitative real-time PCR was performed using the LightCycler TaqMan Master Kit (Roche, Penzberg, Gemany) together with the Universal Probe Library System. RT-PCR primers were designed using the open software “Universal Probe Library Assay Design Center” from Roche [60] and intron-spanning primers selected whenever possible (listed in Table 1). Gene expression was normalized to hypoxanthine phosphoribosyltransferase (HPRT) expression.

### 4.4. Protein Extraction and Immunoblotting

Proteins were extracted in lysis buffer containing 50 mM Tris-HCl, 150 mM NaCl, 0.1% SDS, 1% Triton X-100 (all from Sigma-Aldrich, Taufkirchen, Germany), 1% deoxycholic acid sodium salt (Merck, Darmstadt, Germany), protease, and phosphatase inhibitors (Roche, Penzberg, Germany). Gel electrophoresis and blotting were performed with the XCell SureLock mini-cell electrophoresis system and XCell II blot module with 4–12% gels in MES SDS buffer and PVDF membranes, according to the manufacturer’s protocol (Thermo Fisher Scientific, Waltham, MA, USA) and as described previously [61]. In BRN3A immunoblots using anti-BRN3A (mouse, sc-8429, Santa Cruz Biotechnology Inc., Santa Cruz, CA, USA) antibody, PVDF membranes were denatured after protein transfer by incubating in a dilution series of guanidine thiocyanate (Sigma-Aldrich, Taufkirchen, Germany), as described by Hohenauer et al. [11]. Membranes were then blocked with 5% BSA/PBST and further treated as described by Besch et al. [62]. Primary antibodies were directed against p21 (mouse, sc-6246, Santa Cruz Biotechnology, Santa Cruz, CA, USA); HDAC1 (rabbit, #2062, Cell Signaling Technology, Danvers, MA, USA); HDAC2 (mouse, #5113, Cell Signaling Technology, Denvers, MA, USA); HDAC3 (rabbit, #2632, Cell Signaling); HDAC11 (rabbit, #58442, Cell Signaling Technology, Denvers, MA, USA); FLAG^®^ tag (for detection of myc-DDK-tagged HDAC2) (rabbit, #14793 Cell Signaling Technology, Denvers, MA, USA); and β-actin (mouse, A1978, Sigma-Aldrich, Taufkirchen, Germany), which served as loading control. For detection, HRP-linked horse anti-mouse (#7076, Cell Signaling Technology, Denvers, MA, USA) or HRP-linked goat anti-rabbit (#7074, Cell Signaling Technology, Denvers, MA, USA) secondary antibodies, and Amersham ECL Prime Western Blotting Detection Reagent (GE Healthcare, Little Chalfont, UK), were used according to the manufacturers’ instructions.

### 4.5. DNA Methylation Status Analysis: Bisulfite Conversion, Methylation PCR, and Sequencing

Genomic DNA of untreated HM, Hermes, and melanoma cell lines was extracted using the NucleoSpin^®^ Tissue Kit (Macherey-Nagel, Düren, Germany) according to the manufacturer’s instructions. EpiMark^®^ Bisulfite Conversion Kit (New England Biolabs, Ipswich, MA, USA) was used for bisulfite conversion of 2 µg of genomic DNA following the manufacturer’s protocol. Primers were designed for subsequent methylation-specific PCR to amplify of the genomic region of interest, which contains a putative CpG island, using MethPrimer software (http://www.urogene.org/cgi-bin/methprimer/methprimer.cgi, accessed on 22 January 2019 [23]). These primers bind exclusively to bisulfite-converted DNA in genomic regions adjacent to the putative CpG island within the *BRN3A* promoter region (primer forward: 5′-TTGTTTTTGTTGGTGGTTATTATAAGA-3′; primer reverse: 5′-AATACACTCCTCTAACACCTAAACCC-3′). The region of interest ranging from position −229 bp to −11 bp upstream of the *BRN3A* transcription start site (TSS) was amplified. As PCR template, 250 mg bisulfite-converted DNA was mixed with 5x EpiMark^®^ Hot Start Taq Reaction Buffer (New England Biolabs, Ipswich, MA, USA), 200 µM dNTPs, 0.2 µM Meth primer forward, 0.2 µM Meth primer reverse, and 1.25 units EpiMark^®^ Hot Start Taq DNA Polymerase (New England Biolabs, Ipswich, MA, USA) and amplified by PCR (see Table 2 for cycling protocol). Five µL PCR mix was then separated on a 1.5% agarose gel to check the size of the PCR products. The rest of the PCR mix was purified using the NucleoSpin^®^ Gel and PCR Clean-up Kit (Macherey-Nagel, Düren, Germany), according to the manufacturer’s instructions, in order to remove dNTPs and methylation primers that may interfere with the sequencing reaction. Five µg of purified PCR amplicon was mixed with 0.2 µM sequencing primer (5′-CACTCCTCTAACACCTAAACCC-3′) for Sanger sequencing (custom DNA sequencing service by Eurofins Genomics, Ebersberg, Germany). Sequencing results were compared and analyzed using the program NCBI BLAST^®^ (https://blast.ncbi.nlm.nih.gov/Blast.cgi, accessed on 25 July 2019) [63].

### 4.6. siRNA Transfection

Small interfering RNAs (siRNAs) against HDAC1 (5′-CCGGUCAUGUCCAAAGUAA-3′), HDAC2 (5′-GGAUAUUGGUGCUGGAAAA-3′, referred to as HDAC2 #1), and HDAC3 (5′-CGGUGUCCUUCCACAAAUA-3′) were synthesized and obtained from MWG Eurofins (Ebersberg, Germany). An siRNA against HDAC11 (Ambion Silencer siRNA, assay ID 130749) and a second siRNA against HDAC2 (assay ID 120210, referred to as HDAC2 #2) were purchased from Thermo Fisher Scientific (Waltham, MA, USA). For controls, a siRNA with a random sequence that did not match within the human genome was used [64]. For transfection experiments, cells were seeded at six-well plates one day prior to transfection, as described above. Melanoma cells were transfected at 20 nM siRNA with 1.25 µL Lipofectamine RNAiMAX (Thermo Fisher Scientific, Waltham, MA. USA), and HM and Hermes were transfected with 20 nM siRNA with JetPRIME (Polyplus-Transfection, Illkirch, France), according to the manufacturer’s instructions. Cells were incubated for 48 h and then harvested for protein extraction, as described above.

### 4.7. Ectopic HDAC2 Expression

The melanoma cell lines 1205Lu and WM165 were seeded in six-well plates as described above in Section 4.1 and transfected with 1 µg/well Myc-DDK-tagged human histone deacetylase 2 (HDAC2), transcript variant 1 expression plasmid (#RC224919) or the empty vector pCMV6-Entry (#PS100001, both from OriGene Technologies Inc., Rockville, MD, USA), using FuGENE^®^ 6 Transfection Reagent (Promega, Madison, WI, USA) according to the manufacturer’s instructions. After 48 h, cells were harvested and protein expression of BRN3A and ectopically expressed HDAC2 were determined as described in Section 4.4.

### 4.8. Chromatin Immunoprecipitation and Sequencing (ChIP-Seq), Mapping, and Data Analysis

ChIP-Seq experiments of HDAC2-depleted melanoma cells were conducted as described recently by Kappelmann-Fenzl et al. (2019), with minor modifications [65]. Briefly, 2 × 10^7^ WM9 melanoma cells were transfected with siRNA against HDAC2 (HDAC2 #1) and control siRNA, respectively, in T75 flasks, as described above in Section 4.6. After 24 h, crosslink and lysis of the cells were performed and the resulting nuclei suspensions were sheared to an average DNA target size of approximately 200–700 bp using a ME220 Focused-ultrasonicator (Covaris Inc., Woburn, MA, USA) (duty cycle: 15%, PIP: 75; cycles per burst: 1000, water level: 9; and time: 20 min). After DNA purification; the shearing efficiency was monitored using a 4200 Tape Station (Agilent Technologies, Santa Clara, CA, USA). The ChIP protocol was carried out using an H3K27ac (#ab4729, Abcam, Cambridge, UK)-specific antibody.

Purified DNA obtained from ChIP (10–50 ng) was linked to adapters, amplified by PCR, and sequenced for 50 cycles on a HiSeq 3000/4000 (Illumina, San Diego, CA, USA) according to the manufacturer’s instructions. Bowtie 2 software (version 2.2.7) [66] was used to map the sequence tags of all experiments to the current human reference sequence GRCh38/hg38. For further analysis, only uniquely mapped tags were used, and tag counts were normalized to 10^7^ specifically mapped tags. Mapped ChIP-Seq tags were analyzed using the HOMER software (available at http://homer.ucsd.edu/homer/, accessed on 10 September 2019 [67,68]) previously described by Kappelmann-Fenzl et al. [65]. Peaks were defined at a 1 × 10^−6^ estimated false discovery rate.

### 4.9. Light Microscopy

Microscopic images were taken with an Axiovert 25 light microscope (Carl Zeiss, Jena, Germany) and Axiovert 25 Imaging Software 4.7.2 (Carl Zeiss, Jena, Germany), at 100-fold magnification.

### 4.10. Cell Viability Assay

Cell viability after HDAC inhibition was determined by a resazurin-based fluorimetric assay using the CellTiter-Blue^®^ Cell Viability Assay (Promega, Madison, WI, USA) according to the manufacturer’s instructions. Non-fluorescent resazurin can be reduced to fluorescent resorufin by viable cells, and resorufin fluorescence can be determined (579Ex/584Em) [68]. Briefly, 100,000 cells were seeded in six-well plates and exposed to TSA or DMSO (control) for 6 h, 24 h, and 48 h. After that, 70 µL of CellTiter-Blue^®^ was added to 375 µL medium per well and incubated at 37 °C for 1 h. Then, 100 µL was transferred into a 96-well plate and fluorescence was measured by a CytoFluor™ 2350 fluorometer (Millipore, Schwalbach, Germany). Cell viability was calculated relative to the corresponding DMSO controls.

### 4.11. Statistical Analysis

All experiments were performed in at least three independent experiments, unless otherwise described. Data show mean values ± SD. GraphPad Software v 5.04 (San Diego, CA, USA) was used for all statistical calculations unless otherwise stated. Two groups were compared using the unpaired two-tailed Student’s t-test, and differences were considered significant at a *p*-value of 0.05 or less. All statistical calculations of the ChIP-Seq results were performed using the GraphPad Prism Software (version 8, GraphPad Software Inc., La Jolla, CA, USA), R (version 3.6.1, https://www.r-project.org/), accessed on 10 September 2019 [69], and RStudio (version 1.1.456, RStudio Inc., Boston, MA, USA, http://www.rstudio.com/), accessed on 10 September 2019 [70]. Group comparisons of next-generation sequencing data were made by the non-parametric Wilcoxon test.

## 5. Conclusions

In summary, we have identified acetylation and deacetylation of histones of a distal enhancer element mediated by HDAC2 to regulate gene expression of *BRN3A*. These results highlight the importance of epigenetic regulatory mechanisms of melanoma oncogenes.

## Figures and Tables

**Table 1:** Primers for mRNA quantification by qRT-PCR.

Gene Name	Primer Forward (5′→3′)	Primer Reverse (5′→3′)	Probe No.
HPRT	TGACCTTGATTTATTTTGCATACC	CGAGCAAGACGTTCAGTCCT	73
BRN3A	TTTCCTCCACCCATTCTCTG	ACCCCAGTCCTCAAGGCTA	56
HDAC2	CAGATCGTGTAATGACGGTATCA	CCTTTTCCAGCACCAATATCC	72
CDKN1A	TCACTGTCTTGTACCCTTGTGC	GGCGTTTGGAGTGGTAGAAA	32
SFN	TCGTAGGAATTGAGGAGTGTCC	ACACACACACCCATCTCCAG	5

**Table 2:** Cycling protocol for methylation PCR.

Cycle Step	Temperature	Time	
Initial denaturation	95 °C	60 s	1 cycle
Denaturation	95 °C	30 s	
Annealing	53 °C	30 s	40 cycles
Extension	68 °C	30 s	
Final extension	68 °C	5 min	1 cycle

## Supplementary Materials

The following are available online at https://www.mdpi.com/article/10.3390/ijms23020849/s1.
