# Supplementary material for: HDAC2 Is Involved in the Regulation of BRN3A in Melanocytes and Melanoma
Source: Int J Mol Sci. 2022 Jan 13;23(2):849. doi: 10.3390/ijms23020849 (PMC8778714; doi:10.3390/ijms23020849)
Supplement: Supplementary file 1 [file ijms-23-00849-s001.zip › ijms-1539543-supplementary.pdf]

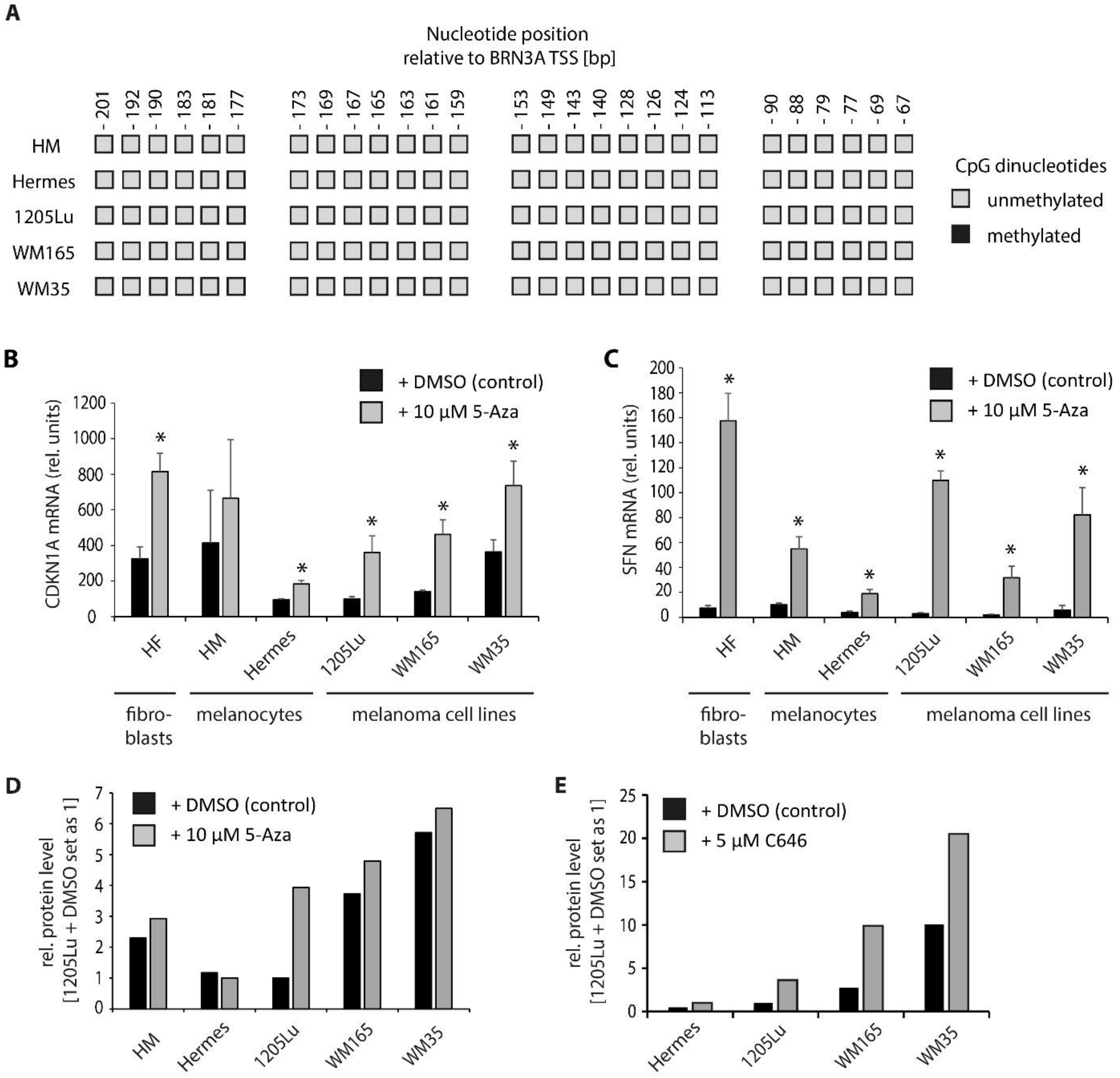

Supplementary Figure S1. Methylation-specific sequencing and analysis of DNMT inhibition efficacy. **(A)** Methylation-specific sequencing of a putative CpG island located within the *BRN3A* promoter region. Genomic DNA of untreated melanocytes (HM, Hermes) and melanoma cell lines (1205Lu, WM165, WM35) was isolated and unmethylated cytosines were converted to uracil by bisulfite conversion. The genomic region from position -229 bp to -11 bp upstream of the *BRN3A* TSS containing a putative CpG island as predicted by the *MethPrimer* software (http://www.urogene.org/cgi-bin/methprimer/methprimer.cgi [23]) was amplified by PCR with primers that bind exclusively to bisulfite-converted DNA. Sequencing of the PCR products revealed no methylation of any CpG dinucleotides in this genomic area. The illustration shows the nucleotide positions -201 bp to -67 bp of potentially methylated cytosines relative to the *BRN3A* TSS. **(B)** Relative gene expression levels of *CDKN1A* coding for the cell cycle regulating protein $p21^{Cip1/Waf1}$ and **(C)** stratifin (*SFN*, coding for 14-3-3 σ protein) in human fibroblasts (HF), melanocytes (HM), Hermes cells and a panel of melanoma cell lines (1205Lu, WM165, WM35) after treatment with 10 µM 5-Aza (+) or DMSO (control, Ø) for 48 h, n=3, *: $p < 0.05$ vs. corresponding DMSO control. Treatment with 5-Aza led to upregulation of p21 and SFN as reported previously [25,27]. **(D)** Densitometric analysis of p21 protein expression normalized to β-actin protein expression, corresponding immunoblot is shown in Figure 1E. **(E)** Densitometric analysis of p21 protein expression normalized to β-actin protein expression, corresponding immunoblot is shown in Figure 2B.

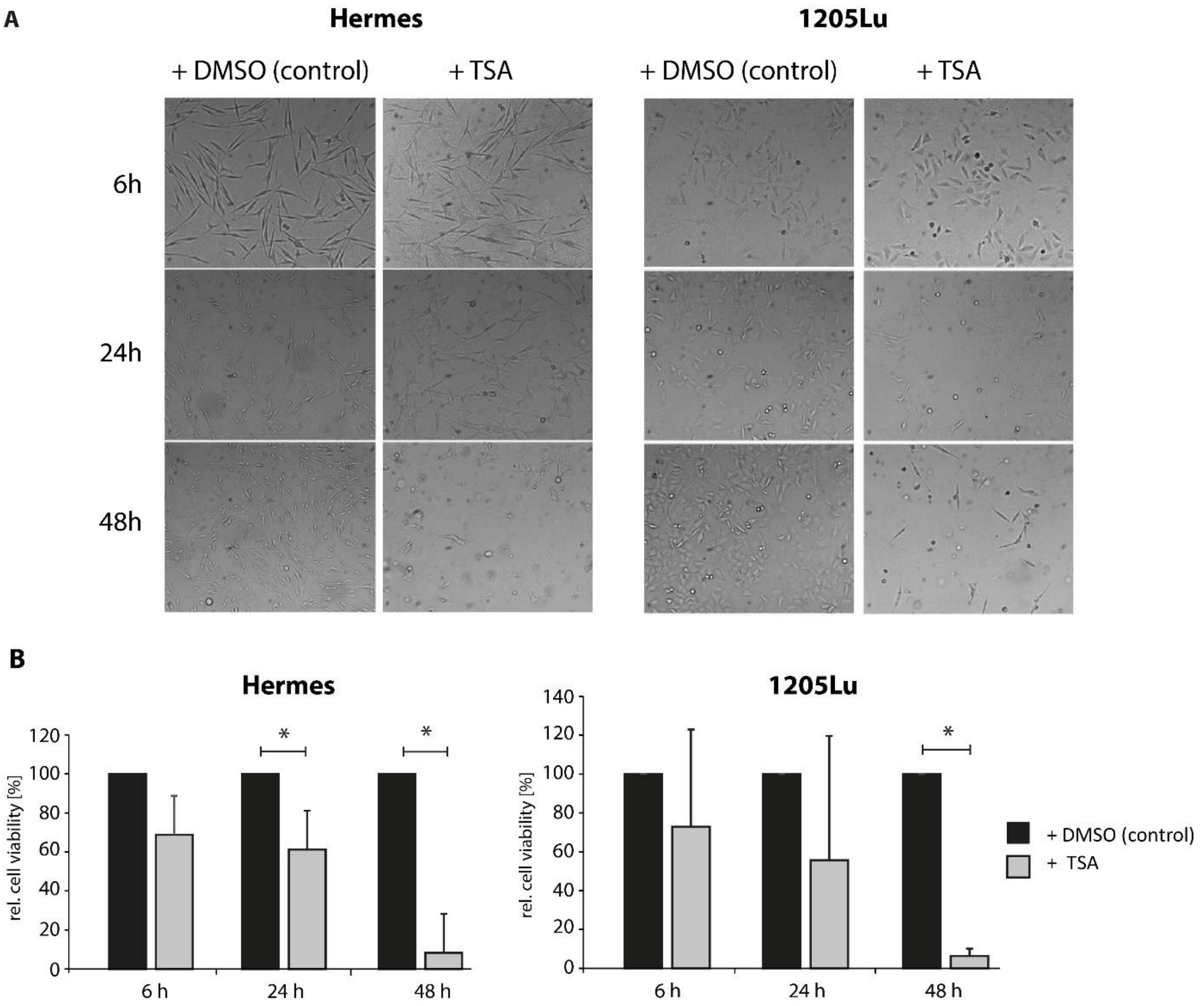

Supplementary Figure S2. Prolonged exposure to TSA induced cell death in Hermes and 1205Lu melanoma cells. **(A)** Microscopic images of Hermes (left) and 1205Lu (right) cells after incubation with 10 µM TSA or DMSO (control) for 6 h, 24 h and 48 h, 100x magnification. **(B)** Relative cell viability determined by CellTiter-Blue® Cell Viability Assay. Hermes (left) and 1205Lu cells (right) were incubated with 10 µM TSA or DMSO (control) for 6 h, 24 h and 48 h. Normalization to corresponding DMSO control (100%), n=3. *: $p < 0.05$.

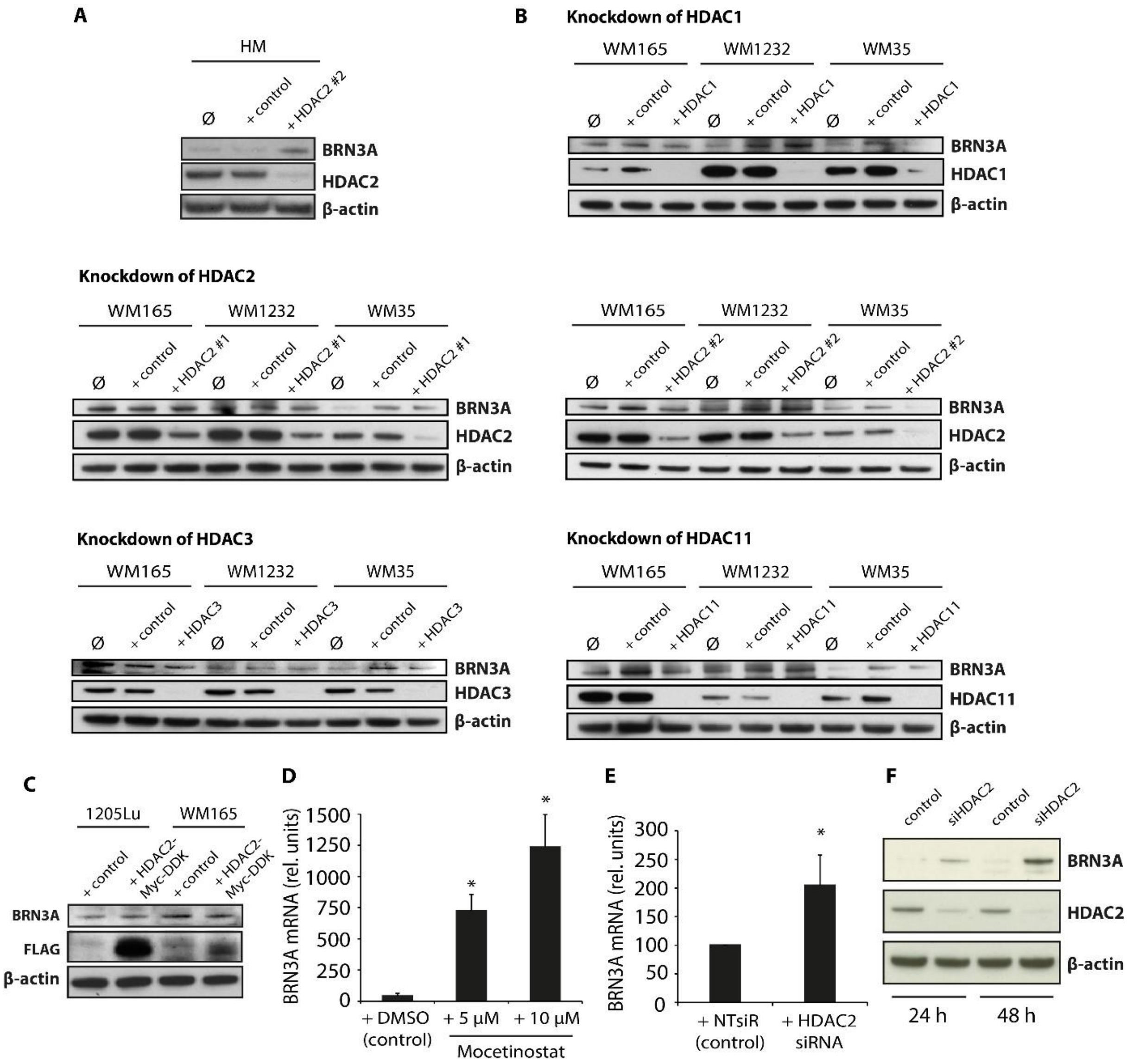

Supplementary Figure S3. (A) HDAC2 inhibition by a second siRNA also induces BRN3A protein expression in melanocytes. Melanocytes were transfected with a second HDAC2-specific siRNA (HDAC2 #2, assay ID: 120210, Thermo Fisher Scientific) or non-targeting control siRNA (control) for 48 h. Ø: transfection reagent only. **(B)** Protein expression of BRN3A as well as HDAC1, HDAC2, HDAC3 and HDAC11 after knockdown of HDAC1, 2, 3, and 11, respectively, in melanoma cells WM165, WM1232 and WM35 after 48 h. HDAC1, HDAC2 #1, HDAC2 #2, HDAC3 and HDAC11: specific siRNAs targeting HDAC1, HDAC2 (#1, #2: two different siRNAs against HDAC2), HDAC3 and 11, respectively; control: non-targeting control siRNA; Ø: transfection reagent only. Representative immunoblots are shown. **(C)** Ectopic expression of HDAC2 in WM9 melanoma cells 48 h after transfection. Cells were transfected with a vector for ectopic HDAC2 containing a Myc and a FLAG® (DDK-) tag or an empty control vector (pCMV6 entry). **(D)** Relative *BRN3A* gene expression levels in WM9 melanoma cells after treatment with 5 µM or 10 µM mocetinostat or DMSO (control) for 24 h, n=3. *: $p < 0.05$ vs. corresponding DMSO control. **(E)** Relative *BRN3A* gene expression levels in WM9 melanoma cells after knockdown of HDAC2, n=3. NTsiR: non-targeting control siRNA. **(F)** BRN3A and HDAC2 protein expression in WM9 melanoma cells 24 h and 48 h after transfection with HDAC2 siRNA (siHDAC2) or non-targeting control siRNA (control).
